# Study on the Effect of Sooty Mould Disease in Tea Plants

**DOI:** 10.3390/plants13162321

**Published:** 2024-08-20

**Authors:** Renjian Liu, Hongmei Liu, Yuyuan Wang, Jiahao Chen, Zihao Qiu, Yanchun Zheng, Binmei Sun, Xindong Tan, Canwei Shu, Shaoqun Liu, Peng Zheng

**Affiliations:** 1 Department of Tea, College of Horticulture, South China Agricultural University, Guangzhou 510642, China; liurj_scau@163.com (R.L.); scau20213137137@stu.scau.edu.cn (H.L.); wangyy@stu.scau.edu.cn (Y.W.); cjhtea@stu.scau.edu.cn (J.C.); scau20222018004@stu.scau.edu.cn (Z.Q.); zhengyanchun@stu.scau.edu.cn (Y.Z.); binmei@scau.edu.cn (B.S.); txd@scau.edu.cn (X.T.); 2 Guangdong Province Key Laboratory of Microbial Signals and Disease Control, College of Plant Protection, South China Agricultural University, Guangzhou 510642, China

**Keywords:** sooty mould (SM) disease, tea plants, transcriptome analysis, *Cladosporium pseudocladosporioides*, effect

## Abstract

Sooty mould (SM) disease affects the growth, development and metabolism of plants and reduces the commodity and economic value of crops. SM disease is one of the important leaf diseases in tea plants. Nonetheless, studies on the effect of SM disease in tea plants are rare. Herein, we found that SM disease disrupted the cell morphology and structure and reduced the contents of caffeine, theanine, and catechins in the mature leaves of tea plants. Transcriptome analysis revealed that SM disease inhibited the biosynthesis of lignin, chlorophyll, catechin, caffeine, and theanine and affected the plant-pathogen interactions in the mature leaves of tea plants by downregulating gene expression. In addition, two fungal isolates, MTzyqA and MTzyqB, were obtained from the mature leaves of diseased tea plants. These strains were identified as *Cladosporium pseudocladosporioides* by mulitgene phylogenetic analysis, and they grew epiphytically on the leaves of tea plants. The biocontrol bacteria JT68, ZGT5, and BX1 had obvious inhibitory effect on MTzyqA and MTzyqB. These results provide a basis for understanding the effect of SM disease in tea plants.

## 1. Introduction

Sooty mould (SM) disease is an important disease in plants and is prevalent in tropical and subtropical regions around the world [1]. It can affect the growth, development, and metabolism of plants and reduce the commodity and economic value of crops [2,3,4]. The symptoms of SM disease are black, sooty and black velvety layer of mycelium, and thick crust-like coatings on the surface of leaves and fruits in severe cases [5]. SM disease can directly inhibit the photosynthesis in plants by reducing photosynthetic rate [6,7]. Fungi responsible for SM disease have diverse life styles, they can be parasites or saprotrophs. Parasitic fungi absorb nutrients from mesophyll cells through haustorium, while saprophytic fungi colonize the leaves of plants and use honeydew secreted by insects or the secretions of plants as a source of nutrition [1,8].

*Cladosporium* species are major pathogens that causes SM disease in many plants. They are typical saprophytic fungi widely distributed in the natural environment, and can be isolated from soil and decaying plant material [9]. *Cladosporium* species are one of the largest and most heterogeneous genera of hyphomycetes, comprising three major species complexes: *C. cladosporioides*, *C. herbarum*, and *C. sphaerospermum* [10,11]. A thick refractive to darkened cladosporioid or coronate scar which is defined as a raised periclinal rim with a central convex dome is the unique structure of conidiogenous loci and conidial hila in the *Cladosporium* genus [10]. The main differences between the three major species complexes lie in morphological characteristics. *C. cladosporioides* species complexes possess regular and feathery colonies with sparse and diffuse or sometimes abundantly formed white aerial mycelia, *C. herbarum* species complexes are characterized by the formation of chlamydospores, and the conidia of *C. sphaerospermum* species complexes are smaller than those of the other two groups and have no septa [11]. *Cladosporium* species are common dominant endophytic fungal genuses of many plants, including common bean, mangrove, tea plants, and *Salvia miltiorrhiza* [12,13,14]. However, *Cladosporium* species have been also identified as pathogens in plants. *C. cladosporioides* species complexes caused SM disease in pitaya [4] and fruit rot in raspberries, grapes, and sweet pepper [15,16]. *C. cladosporioides* species complexes could also cause seed decay of tobacco and blossom blight in strawberries [1,17].

Tea plants (*Camellia sinensis*), an evergreen shrub in the *Theaceae* family, is one of the most important industrial crops [18]. In China, the tea planting area is approximately 51 million acres, and the tea production is up to 334.21 tons. The leaves of tea plants contain abundant metabolites, including theanine, catechins, volatiles, and caffeine. These metabolites have different flavours and tastes in tea infusions [19,20]. The tender leaves of tea plants are covered with white trichomes through the surface, and they are often used to process into black and green tea. While the mature leaves of tea plants are relatively smooth without trichome characteristic, and they are generally used to process into Pu’er and oolong tea [19,20]. Thus, leaves are the economic and utilized parts of tea plants and are processed into various kinds of tea (fermentation degree and processing technology are different) [21]. However, tea production is severely affected by various biotic factors, especially pathogens (viruses, fungi and bacteria) [22]. Leaf disease severely affect the physiological functions, biochemical components, and production and quality of tea plants [3,21,23]. Among them, SM disease is an important leaf disease in tea plants. In recent years, as pests and the weather of high temperature and high humidity increasing, a large-scale outbreak of SM disease occurred in the tea-producing area of Metuo County, Nyingchi City, Tibet Autonomous Region, China, which greatly affected the development of local tea industry [24]. However, studies on SM disease in tea plants are rare, and only one study revealed that exogenous melatonin could reduce the damage caused by SM disease in tea plants [25].

In this study, the effects of SM disease on the morphology, structure, and quality of tea plant leaves were analysed. The molecular mechanism of SM disease in tea plants was preliminarily analysed via transcriptome sequencing. Moreover, we preliminarily isolated and identified the fungi responsible for SM disease in tea plants, and performed biocontrol experiments with bacteria. This study provides a basis for understanding the effect of SM disease in tea plants.

## 2. Results

### 2.1. Effects of SM Disease on the Morphology, Structure and Composition of the Mature Leaves of Healthy and Diseased Tea Plants

**Figure 1:**
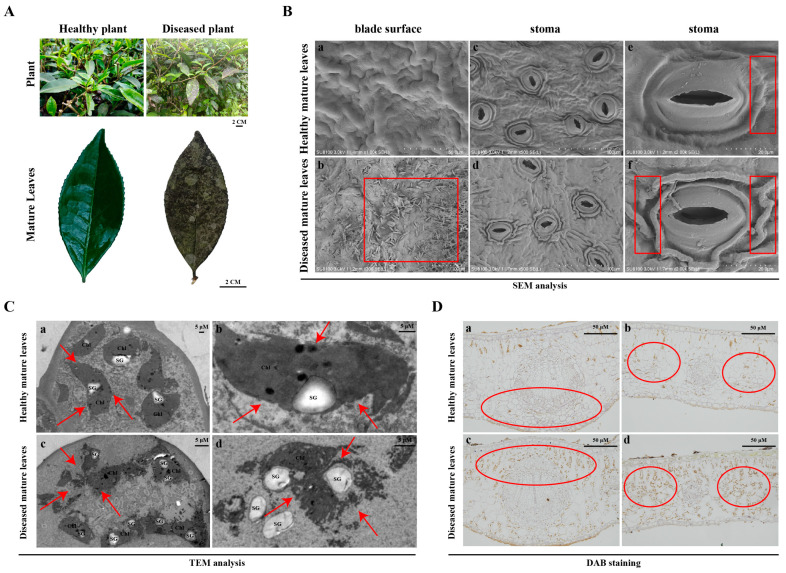
Plant phenotype, cell morphology and structure of the mature leaves of healthy and diseased tea plants. (**A**). Plant phenotype of the mature leaves of healthy and diseased tea plants. Bar = 2 cm. (**B**). SEM analysis of the mature leaves of healthy and diseased tea plants. a,b: blade surface of healthy mature leaves and diseased mature leaves; c–f: stoma of healthy mature leaves and diseased mature leaves. The red box indicated the symptoms of the mature leaves after SM infection. (**C**). TEM analysis of the mature leaves of healthy and diseased tea plants. a,b: cell structure of healthy mature leaves; c,d: cell structure of diseased mature leaves. Bar = 5 μM. The red arrow indicated the symptoms of the mature leaves after SM infection. Chl: chloroplast; SG: starch grain. (**D**). DAB staining of the mature leaves of healthy and diseased tea plants. a,b: DAB staining of healthy mature leaves; c,d: DAB staining of diseased mature leaves. Bar = 50 μM. The red circle indicated the symptoms of the mature leaves after SM infection.

To understand the effects of SM disease on the cell morphology and structure of tea plant leaves, the mature leaves of healthy and diseased tea plants were observed by scanning electron microscopy (SEM) analysis, transmission electron microscopy (TEM) analysis, periodic acid-schiff (PAS) staining, toluidine blue O (TBO) staining, and 3,3′-diaminobenzidine (DAB) staining. Compared with those of healthy tea plants, the mature leaves of diseased tea plants after SM infection were clearly covered with plaque made up of a reticular dark brown layers of mecelium; however, there was very little plaque on the tender leaves of tea plants (Figure 1A). The leaves became dry with light discolouration and fell from tea plants as a consequence of SM disease. SEM analysis revealed that compared with those of healthy tea plants, the paraxial surfaces of the mature leaves of diseased tea plants after SM infection were covered with a large amount of mycelium and spores, and the guard cells beside the stomata had many folds (Figure 1B). TEM analysis revealed that compared with those of healthy tea plants, the chloroplast boundaries of the mature leaves of diseased tea plants after SM infection became more blurred and dissolved, most chloroplasts were mixed together, and the number of starch particles increased (Figure 1C). In addition, mycelium and spores were not found in the interior of the mature leaves of diseased tea plants after SM infection (Figure 1C). The results of PAS, TBO and DAB staining indicated that compared with those of healthy tea plants, the polysaccharides and proteins of the mature leaf cells of diseased tea plants significantly decreased (Supplementary Figure S1), while the Hydrogen peroxide (H_2_O_2_) content significantly increased (Figure 1D). These results suggested that SM disease altered the cell morphology, structure and composition of the mature leaves of tea plants.

### 2.2. Effects of SM Disease on the Quality Components of the Tender and Mature Leaves of Healthy and Diseased Tea Plant

**Figure 2:**
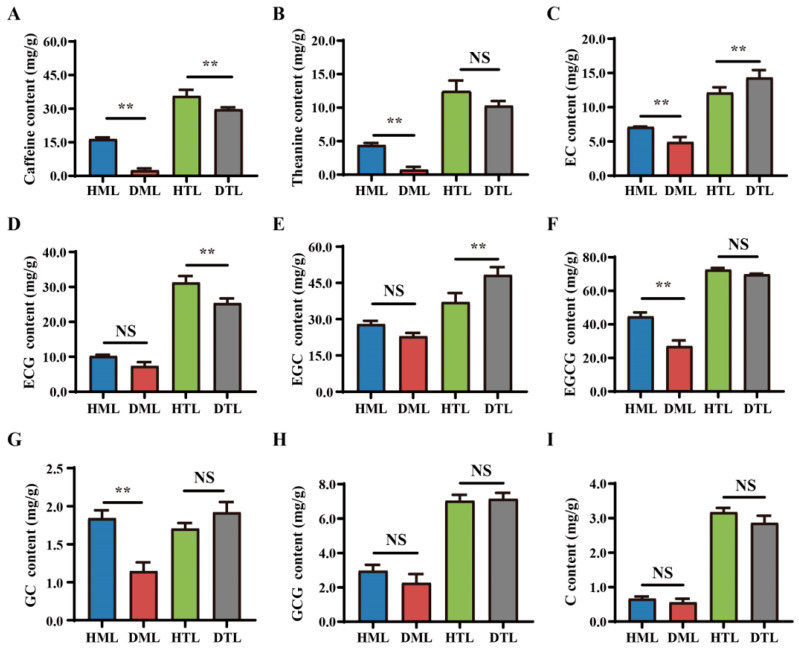
Determination of caffeine (**A**), theanine (**B**), and catechin (**C**–**I**) contents of the tender and mature leaves of healthy and diseased tea plants. HML: healthy mature leaves; DML: diseased mature leaves; HTL: healthy tender leaves; DTL: diseased tender leaves. The data were presented as the Means ± SDs (n = 9), and asterisks indicated significant differences compared with ‘HML’ or ‘HTL’ (NS, not significant; **, *p* < 0.01; Student’s *t* test).

To study the effect of SM disease on the quality of tea plant leaves, the contents of caffeine, theanine and catechins of the tender and mature leaves of healthy and diseased tea plants were determined. Compared with those of healthy tea plants, the caffeine content of the tender and mature leaves and theanine content of the mature leaves significantly decreased in diseased tea plants (Figure 2A,B). The contents of seven catechins, including catechin (C), epicatechin (EC), epigallocatechin (EGC), catechin gallate (CG), epicatechin gallate (ECG), gallocatechin gallate (GCG), and epigallocatechin gallate (EGCG), were determined in the leaves of tea plants. Compared with those of healthy tea plants, no significant change was detected in C and GCG contents of the tender and mature leaves of diseased tea plants (Figure 2H,I); the contents of EC, EGCG and GC of the mature leaves significantly decreased in diseased tea plants (Figure 2C,F,G), while the contents of ECG and EGC did not change (Figure 2D,E). The contents of EC and EGC of the tender leaves significantly increased in diseased tea plants (Figure 2C,E), the content of ECG significantly decreased (Figure 2D), and the contents of EGCG and GC remained unchanged (Figure 2F,G). These results suggested that SM disease reduced the content of some quality components of tea plant leaves.

### 2.3. Differentially Expressed Gene (DEG) Analysis of the Mature Leaves of Healthy and Diseased Tea Plants

**Figure 3:**
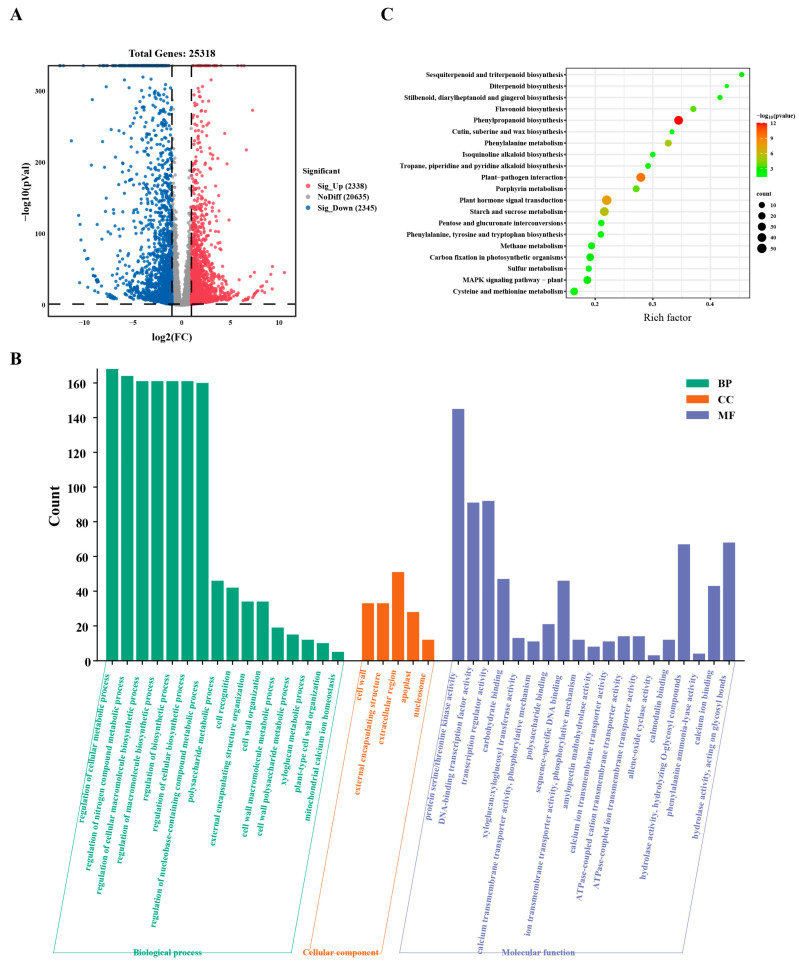
Differentially Expressed Gene (DEG), Gene Ontology (GO), and Kyoto Encyclopedia of Genes and Genomes (KEGG) enrichment analysis of the mature leaves of healthy and diseased tea plants. (**A**). Statistical analysis of the number of DEGs of the mature leaves of healthy and diseased tea plants. (**B**). GO enrichment analysis of DEGs of the mature leaves of healthy and diseased tea plants. (**C**). KEGG enrichment analysis of DEGs of the mature leaves of healthy and diseased tea plants.

To preliminarily understand the molecular mechanism of SM disease in tea plants, the mature leaves of healthy and diseased tea plants were used for RNA sequencing (RNA-seq) analysis. The results of principal component analysis (PCA) and correlation analysis indicated that the data were of high quality and repeatable (Supplementary Figure S2). Compared with those of the mature leaves of healthy tea plants, 2338 up-regulated genes and 2345 down-regulated genes were found in the mature leaves of diseased tea plants (Figure 3A). Gene Ontology (GO) enrichment analysis of the DEGs revealed that the biological process and molecular function terms were enriched, including the regulation of some metabolic processes, the activity of protein serine/threonine kinases, and transcription factors (Figure 3B). Kyoto Encyclopedia of Genes and Genomes (KEGG) enrichment analysis of the DEGs revealed that the DEGs were enriched in phenylpropanoid biosynthesis, plant-pathogen interactions, and plant hormone signal transduction pathways (Figure 3C).

### 2.4. Expression Analysis of DEGs Involved in Key Pathways of the Mature Leaves of Healthy and Diseased Tea Plants

**Figure 4:**
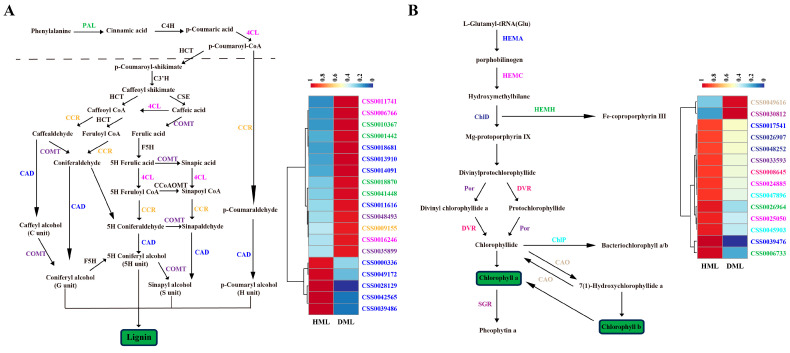
Expression analysis of DEGs involved in lignin and chlorophyll biosynthesis pathways in the mature leaves of healthy and diseased tea plants. (**A**). Expression analysis of DEGs involved in lignin biosynthesis pathway in the mature leaves of healthy and diseased tea plants. (**B**). Expression analysis of DEGs involved in chlorophyll biosynthesis pathway in the mature leaves of healthy and diseased tea plants. The compound names were shown below each arrow. The abbreviations beside the arrows indicated the enzymes catalysing the transfer. HML, healthy mature leaves; DML, diseased mature leaves.

**Figure 5:**
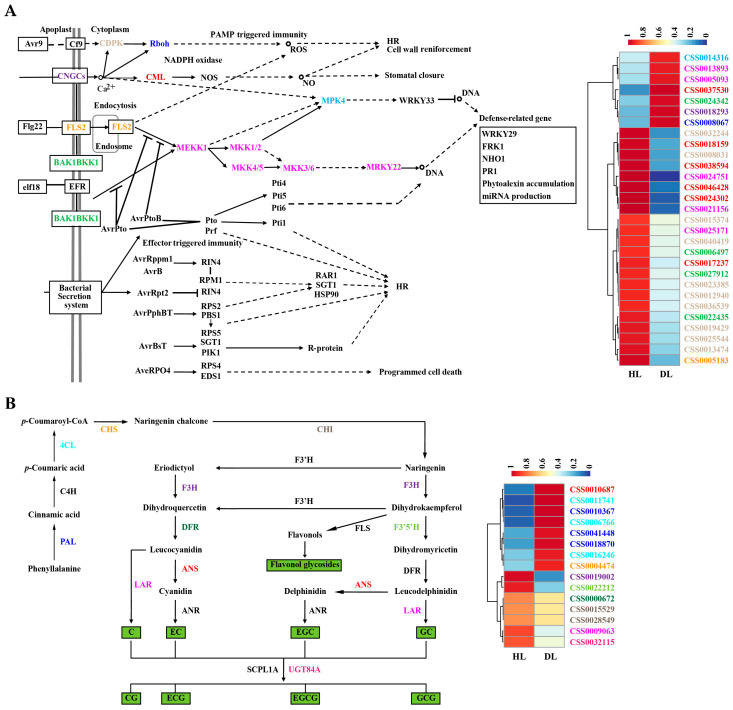
Expression analysis of DEGs involved in plant–pathogen interactions and catechin biosynthesis pathways in the mature leaves of healthy and diseased tea plants. (**A**). Expression analysis of DEGs involved in the plant-pathogen interaction biosynthesis pathway in the mature leaves of healthy and diseased tea plants. (**B**). Expression analysis of DEGs involved in the catechin biosynthesis pathway in the mature leaves of healthy and diseased tea plants. The compound names were shown below each arrow. The abbreviations beside the arrows indicated the enzymes catalysing the transfer. HML, healthy mature leaves; DML, diseased mature leaves.

To further understand the molecular mechanism of SM disease in tea plants, the expression of DEGs involved in several key pathways, including lignin, chlorophyll, catechin, caffeine and theanine biosynthesis and plant–pathogen interaction pathways, were analysed. In the lignin biosynthesis pathway, although the expression of many DEGs was upregulated in the mature leaves of diseased tea plants, the expression of the key downstream biosynthesis-related gene *CAD* was downregulated (Figure 4A). In the chlorophyll biosynthesis and plant-pathogen interaction pathways, the expression of most DEGs was downregulated in the mature leaves of diseased tea plants (Figure 4B and Figure 5A). In addition, in the catechin biosynthesis pathway, the expression of the key downstream biosynthetic genes *LAR* and *UGT84A* decreased in the mature leaves of diseased tea plants (Figure 5B), and the expression of DEGs involved in caffeine and theanine biosynthesis pathways also decreased in the mature leaves of diseased tea plants (Supplementary Figure S3). These results suggested that SM might inhibit lignin, chlorophyll, catechin, caffeine and theanine biosynthesis and plant-pathogen interaction pathways in the mature leaves of tea plants by downregulating gene expression.

### 2.5. Isolation, Molecular Identification, and Pathogenicity Analysis of Pathogenic Fungi

**Figure 6:**
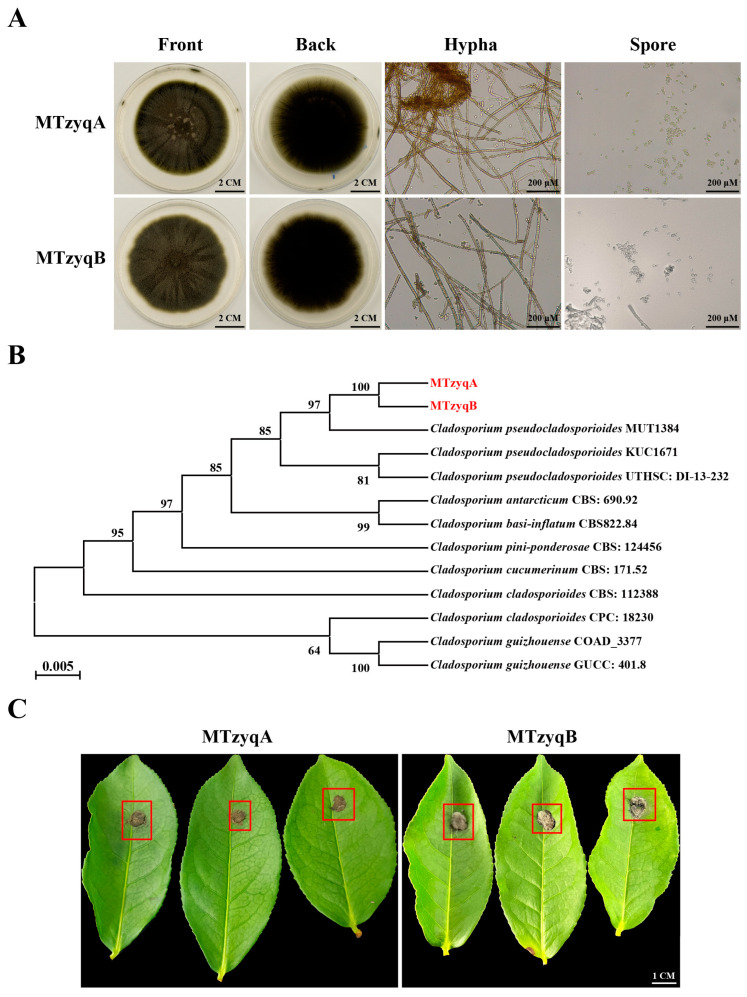
Isolation, molecular identification, and pathogenicity analysis of pathogenic fungi. (**A**). Morphological observation of fungal isolates from the mature leaves of diseased tea plants. Bar = 200 μM or 2 cm. (**B**). Phylogenetic evolutionary tree analysis of fungal isolates. (**C**). Pathogenicity analysis of fungal isolates. Bar = 1 cm. The red box indicated the symptoms of the leaves after SM infection.

To identify the causal agent of SM disease in tea plants, two fungal isolates, MTzyqA and MTzyqB, were retrieved from the mature leaves of disease tea plants with typical black coal seam symptoms, which refered to plaque made up of a reticular dark brown layers of mycelium. Morphological observation revealed that the colonies of MTzyqA and MTzyqB were olive-coloured, black velvety layer of mycelium in shape, and radiated outwards (Figure 6A). Microscopically, the mycelia of MTzyqA and MTzyqB were light olive-coloured and less branched, and their spores were light olive-coloured and subglobular (Figure 6A). The results of multigene combined phylogenetic tree showed that MTzyqA and MTzyqB clustered in a branch with *Cladosporium pseudocladosporioides* MUT1384 (Figure 6B). The results of pathogenicity assay showed that four days after inoculation, MTzyqA and MTzyqB showed slightly diffuse growth and they grew epiphytically on the leaves of tea plants (Figure 6C).

### 2.6. Biocontrol Bacterial Sensitivity of Pathogenic Fungi

**Figure 7:**
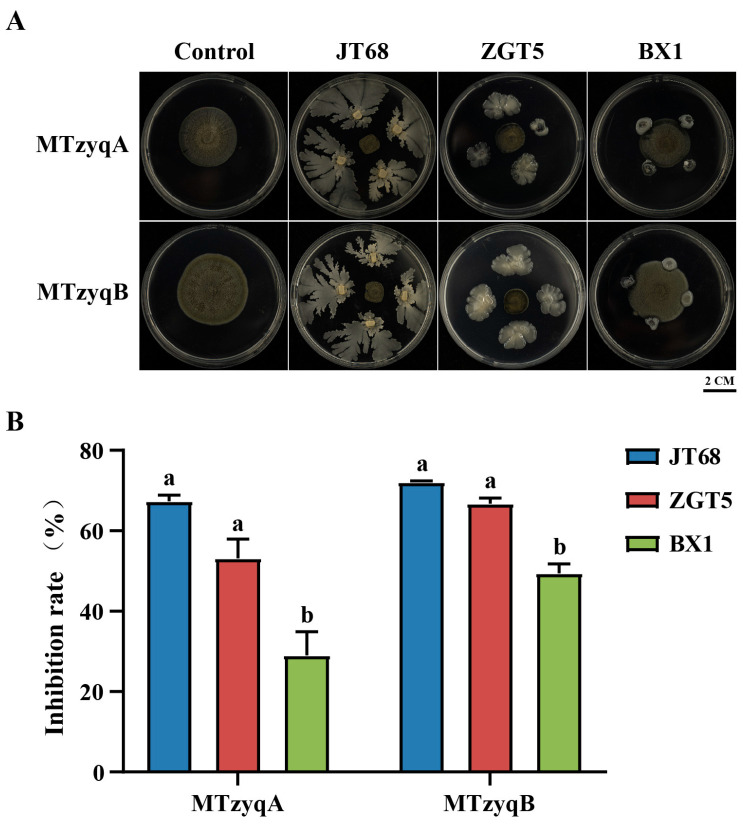
Biocontrol bacterial sensitivity of pathogenic fungi. (**A**). Antagonistic effect of JT68, ZGT5, and BX1 against fungal isolates. Bar = 2 cm. (**B**). Inhibition rates of fungal isolates by JT68, ZGT5, and BX1. The data were presented as the Means ± SDs (n = 9), different letters in the figures indicated significantly different values, and statistically significant differences were determined by One-way analysis of variance (ANOVA) followed by Tukey’s test (*p* < 0.05).

To explore the function of biocontrol bacteria on MTzyqA and MTzyqB, a fungi-bacteria confrontation culture assay was conducted. The results revealed that three biocontrol bacteria, JT68, ZGT5, and BX1, had obvious inhibitory effect on MTzyqA and MTzyqB (Figure 7A). Among them, the inhibitory effect of JT68 and ZGT5 on MTzyqA and MTzyqB were significantly greater than that on BX1 (Figure 7B).

## 3. Discussion

SM disease is an important disease in plants that affects plant growth, development and metabolism [6]. Recently, SM disease has been detected in tea-producing area of Metuo County, Nyingchi City, Tibet Autonomous Region, China, and has severely affected the development of local tea industry [24]. However, reports on SM disease are rare in tea plants. Hence, exploring the effect and molecular mechanism of SM disease is useful for the production and breeding of tea plants.

Chloroplasts in leaves are the main sites of photosynthesis in plants. The leaves of plants are in direct contact with the external environment for a long time, and they are inevitably affected by biotic and abiotic stresses [26]. Pathogenic bacteria and fungi can destroy the cell morphology and structure of leaves and inhibit their growth and development. In *Prunus davidiana* and tobacco, the chloroplast structure of the leaves was disrupted, and the chlorophyll content and photosynthetic rate decreased after pathogen infection [27,28]. In tea plants, blister blight caused deformed growth of the leaves, manifested as swelling and distortion [29,30]. In this study, the cell morphology, structure and composition of the mature leaves of healthy and diseased tea plants were observed via SEM and TEM analysis, and PAS, TBO and DAB staining. After SM infection, the mature leaves of diseased tea plants were obviously covered with plaque made up of a reticular dark brown layers of mecelium, which was not found in the tender leaves of tea plants (Figure 1A). It might be that the trichomes on the tender leaves of tea plants reduced SM infection. SEM analysis revealed that the paraxial surface of the mature leaves of diseased tea plants was covered with a large amount of mycelium and spores (Figure 1B). In walnut and *Lagerstroemia indica*, the mycelium of SM disease did not invade the leaf tissues of diseased plants [31]. TEM analysis revealed that chloroplasts dissolved and starch granules increased in the mature leaves of diseased tea plants (Figure 1C), which was similar to previous studies [25]. These results indicated that the pathogen grew epophytically on the surface of the leaves instead of growing inside. It formed the plaque made up of a reticular dark brown layers of mycelium on the surface of the leaves of tea plants, which hindered the photosynthesis of the mature leaves, thus affecting the interior leaf contents.

The results of PAS, TBO and DAB staining indicated that the polysaccharide and protein contents of the mature leaf cells significantly decreased while the H_2_O_2_ content significantly increased in the mature leaves of diseased tea plants (Supplementary Figure S1 and Figure 1D). SM disease disrupted the cell morphology, structure and composition of the mature leaves of tea plants. Catechins, theanine and caffeine are the main characteristic compounds in tea plants and affect the taste and quality of tea [32,33]. In this study, after SM infection, the caffeine content of the tender and mature leaves and theanine content of the mature leaves significantly decreased in diseased tea plants (Figure 2A,B). Moreover, the content of ECG of the tender leaves significantly decreased in diseased tea plants, and the contents of EC, GC and EGCG of the mature leaves significantly decreased (Figure 2). These results indicated that SM disease reduced the content of some quality components of tea plant leaves.

RNA-seq has been widely used to study the mechanism of plant–pathogen interactions in plants, including rice [34], *Arabidopsis thaliana* [35], grape [36], and tobacco [37]. The effects of several diseases have been studied in tea plants via RNA-seq analysis. The expression of hemiterpenes and cell wall biosynthesis genes increased after infection with blister blight in tea plants [38]. After anthracnose infection in tea plants, the DEGs were mainly involved in plant hormone biosynthesis and signal transduction, plant-pathogen interactions and secondary metabolism biosynthesis pathways [22]. In this study, after SM infection, the DEGs of the mature leaves of disease tea plants were enriched in the phenylpropanoid biosynthesis, plant-pathogen interaction and plant hormone signal transduction pathways (Figure 3). The expression of key downstream biosynthesis-related gene *CAD* in lignin biosynthesis pathway and most DEGs in chlorophyll biosynthesis and plant–pathogen interaction pathways were downregulated in the mature leaves of diseased tea plants (Figure 4 and Figure 5A). These results suggested that SM disease might affect the photosynthesis and disease resistance of tea plants by disrupting the cell wall and chlorophyll biosynthesis of the leaves. In addition, the expression of key downstream biosynthetic genes *LAR* and *UGT84A* in catechin biosynthesis pathway and the DEGs in caffeine and theanine biosynthesis pathways were downregulated in the mature leaves of diseased tea plants (Figure 5B and Supplementary Figure S3), which were consistent with the trend of the contents of catechins, theanine and caffeine in the mature leaves of disease tea plants. To a certain extent, the difference at gene expression was reflected by the difference of the chemical quantities in some quality components. These results revealed that SM disease reduced the contents of catechin, caffeine and theanine of tea plant leaves by downregulating the expression of related biosynthetic genes.

SM disease is an important leaf disease in tea plants, but its pathogen has not been identified. In this study, two fungal isolates, namely MTzyqA and MTzyqB, were obtained from the mature leaves of diseased tea plants with typical black coal seam symptoms, which refered to plaque made up of a reticular dark brown layers of mycelium (Figure 6A). The morphology of these strains is highly similar to that of *C. pseudocladosporioides* reported in previous studies [39]. Multigene combined phylogenetic tree analysis showed that MTzyqA and MTzyqB clustered in a branch with *Cladosporium pseudocladosporioides* MUT1384 (Figure 6B). Moreover, the growth of MTzyqA and MTzyqB slightly increased four days after inoculation in the mature leaves of tea plants (Figure 6C). SM disease in that area may be caused by multiple factors and specific environmental factors conditions. So, a black velvety layer of mycelium as the symptoms from the original mature leaves of diseased tea plants was not found in the inoculation leaves. These results suggested that MTzyqA and MTzyqB were *C. pseudocladosporioides*, which might be pathogens of SM disease; however, their pathogenicity needed further study. The prevention and treatment of SM disease has always been a challenge. Exogenous melatonin could reduce the severity of SM disease in tea plants [25]. However, chemical control might result in chemical residue. Therefore, biological control technology with environmental protection and sustainable development characteristics is urgently needed. JT68, a biocontrol bacterium that belongs to *Bacillus amyloliquefaciens*, can significantly inhibit anthracnose in tea plants [40]. In this study, JT68 and two newly biocontrol bacteria ZGT5 and BX1 (unpublished) that identified by our research group were used for fungi-bacterial confrontation culture assays. JT68, ZGT5 and BX1 could significantly inhibit the growth and diffusion of MTzyqA and MTzyqB (Figure 7), which suggested that biocontrol bacteria have certain biological control potential for SM disease in tea plants. This finding will be helpful for providing useful means for the prevention and treatment of SM disease in tea plants.

## 4. Materials and Methods

### 4.1. Plant Materials

The tea plant variety ‘Zhuyeqi’ was planted in a tea-producing area (29°32′ N, 95°33′ E) in Yarang village, Metuo County, Nyingchi City, Tibet Autonomous Region, China. The tender (one bud and two leaves) and mature leaves of healthy and diseased (with typical black coal seam symptoms: plaque made up of a reticular dark brown layers of mycelium) plants were collected, and three technical replicates of each biological replicate and three biological replicates were included for all samples. All the samples were placed in liquid nitrogen and then stored at −80 °C for further experiments.

### 4.2. SEM Analysis

To visualize the growth process of the pathogenic fungi of SM disease and the morphological changes of tea plant leaves after SM infection, the mature leaves of healthy and diseased plants were collected and observed under an SEM. The samples were prepared following the methods previously described by Sun et al. [41]. The samples were immobilized with a 0.1 M phosphate buffer (pH7.4), dehydrated with alcohol and isoamyl acetate, and dried in a critical point dryer. Subsequently, the prepared specimens were coated with gold and observed using a JSM-6510LV electron microscope (JEOL, Tokyo, Japan) at 15 kV.

### 4.3. TEM Analysis

To observe the effect of SM disease on tea plant leaves, the mature leaves of healthy and diseased plants were collected, and they were sliced into small segments as described previously [31]. The samples were immobilized with a 0.1 M phosphate buffer (pH 7.4), and dehydrated with alcohol and acetone. Subsequently, the samples were permeated with acetone and embedding agent, and sliced in ultra-thin microtome. The sections were stained with uranyl acetate and lead citrate, and observed under a TEM (Jeol Jem 2100f, Tokyo, Japan) [42].

### 4.4. DAB, PAS, and TBO Staining

To further observe the effect of SM disease on tea plant leaves, the mature leaves of healthy and diseased plants were collected for DAB, PAS and TBO staining. The samples were prepared into paraffin sections, which were then stained and observed using a microscope. The DAB staining assay was performed according to the method described by Daudi and O’Brien [43], the PAS staining assay was performed according to the method described by Chawla et al. [44], and the TBO staining assay was performed according to the method described by Qu et al. [45].

### 4.5. Determination of the Contents of Caffeine, Theanine and Catechins

To observe the effect of SM disease on the main quality components of tea plant leaves, the tender and mature leaves of healthy and diseased plants were collected. The contents of caffeine, theanine and catechins were determined using a Waters Alliance Series HPLC system (Waters Technologies, Milford, MA, USA) according to the methods described by our research group [46].

### 4.6. RNA Extraction, Library Construction, RNA-seq, and RNA-seq Data Analysis

Total RNA from the mature leaves of healthy and diseased plants was isolated and extracted using an Eastep^®^ Super Total RNA Extraction Kit (LS1040, Promega, Beijing, China). Library construction was performed according to the method described by Yu et al. [47]. RNA-seq was conducted using an Illumina NovaSeq 6000 instrument by Megg Gene Technology Co. (Guangzhou, China). The raw reads were filtered using Fastp v0.23.4 software with the default parameters by removing the adaptor and low-quality sequences [48]. RNA differential expression analysis between two different groups was performed by DESeq2 [49] and the differentially expressed genes (DEGs) represented genes with a fold_change ≥ 2 and a probability (*p*) value < 0.05. The enrichment analysis of DEGs (*p* < 0.05) was performed using the GO and KEGG databases (http://www.geneontology.org/; https://www.kegg.jp/kegg/; accessed on 1 June 2024). Heatmaps showing the gene expression profiles of the mature leaves of healthy and diseased plants were drawn by R language.

### 4.7. Isolation, Culture, and Morphological Observation of Pathogenic Fungi

Single spore isolation was conducted according to the method described by Chomnunti et al. [6]. We collected the blackened or sooty coating from the symptomatic mature leaves of diseased tea plants by a flamed sterile needle. The collected samples were diluted and placed on potato dextrose agar (PDA) solid medium and incubated in the dark at 25 °C until they started to germinate. After incubation, the single germinated spore was observed using a dissecting microscope. Subsequently, the germinated spores were transferred onto new PDA plates and incubated for 5 days at 25 °C for phenotypic observation. The morphological characteristics of the fungal isolates were observed using a microscope (Biological Microscope BX53, OLYMPUS, Japan).

### 4.8. Molecular Identification of Pathogenic Fungi

To conduct the molecular identification of pathogenic fungi, DNA was extracted using a plant genomic DNA extraction kit (Tiangen Biotech, Beijing, China) from the fungal isolates according to the methods described by Thangaraj et al. [50]. Three different genomic DNA regions of the fungal isolates, including the internal transcribed spacer region (ITS), partial translation elongation Factor 1-alpha (EF1-α) gene, and actin (ACT) gene, were conducted to amplify by Polymerase Chain Reaction (PCR) using the primer pairs ITS1/ITS4 [51], EF1-α688F/1251R [52], and ACT512F/ACT783R [53], respectively. The reference sequences of ITS, ACT and EF1-α in *Cladosporium* species) were obtained from National Center for Biotechnology Information (NCBI), and the corresponding information was listed in Supplementary Table S1. Maximum likelihood (ML) trees were generated using the combined ITS, ACT, and EF1-α datasets via MEGA X with 1000 bootstrap replications.

### 4.9. Pathogenicity Test

The mature leaves of tea plant variety ‘Zhuyeqi’ were used for in vitro inoculation. The fungus was re-isolated from the infected leaves and cultured on the PDA solid medium for 7 days. The pathogenicity tests of two representative fungal isolates were conducted using the wound-inoculation method [22]. The wound was scraped on the tea plant leaves using a inoculating ring, and then a mold cake with a diameter of about 5 mm was placed on the wound of tea plant leaves. The inoculated leaves were placed in an incubator with constant temperature (25 °C) and humidity (70%). The disease incidence was observed four days after inoculation, and the pathogenicity tests was independently repeated three times.

### 4.10. Biocontrol Bacterial Sensitivity Assay

The effectiveness of bacteria as potential biocontrol agents (BCAs) was evaluated in vitro by the dual culture test on agar medium according to the method describrf by Jia et al. [54]. Three biocontrol bacteria JT68 (*Bacillus amyloliquefaciens*), ZGT5 (unpublished) and BX1 (unpublished) used for the assay were isolated and preserved by our research group. The inhibition rate of pathogenic fungi was determined ten days after co-culture and the test was independently repeated three times. Inhibition rate (%) = [(diameter of control fungus-diameter of fungus in co-culture pathogen)/(diameter of control fungus)] × 100.

## 5. Conclusions

Overall, these findings revealed that SM disease disrupted the cell morphology, structure and composition and reduced the content of some quality components of tea plant leaves. SM disease inhibited lignin, chlorophyll, catechin, caffeine and theanine biosynthesis and plan-pathogen interaction pathways in the mature leaves of tea plants by downregulating gene expression. *C. pseudocladosporioides* might be the causal agent of SM disease in tea plants. In addition, the biocontrol bacteria JT68, ZGT5, and BX1 possessed certain biological control potential for SM disease in tea plants.

## Supplementary Materials

The following are available online at https://www.mdpi.com/article/10.3390/plants13162321/s1, Figure S1: PAS and TBO staining of the mature leaves of healthy and diseased tea plants, Figure S2: PCA and correlation analysis of transcriptome data. Figure S3: Expression analysis of DEGs involved in caffeine and theanine biosynthesis pathways in the mature leaves of healthy and diseased tea plants; Table S1: Information of the species gene used for phylogenetic analysis.
