# Supplementary material for: Study on the Effect of Sooty Mould Disease in Tea Plants
Source: Plants (Basel). 2024 Aug 20;13(16):2321. doi: 10.3390/plants13162321 (PMC11359017; doi:10.3390/plants13162321)
Supplement: Supplementary file 1 [file plants-13-02321-s001.zip › Supplementary Figure S.pdf]

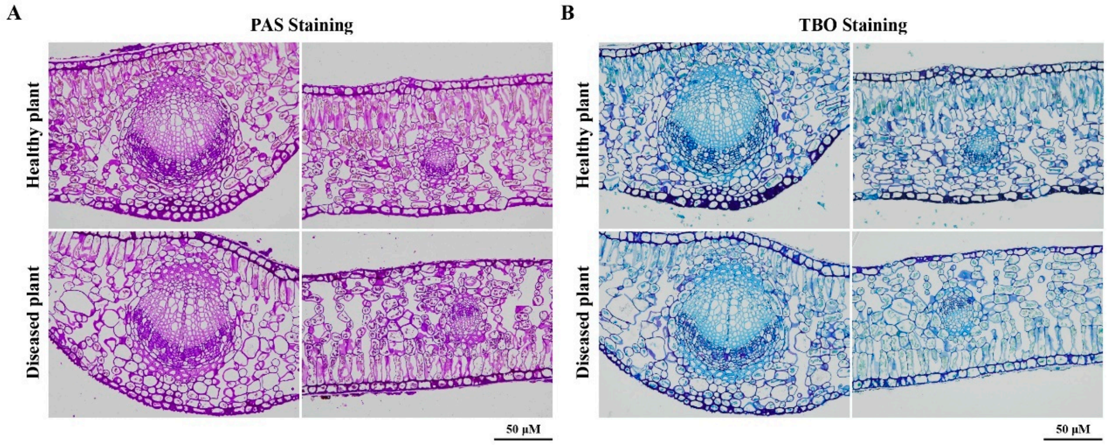

Figure S1 PAS and TBO staining of the mature leaves of healthy and diseased tea plants. A. PAS staining of the mature leaves of healthy and diseased tea plants. B. TBO staining of the mature leaves of healthy and diseased tea plants. Bar = 50 μM.

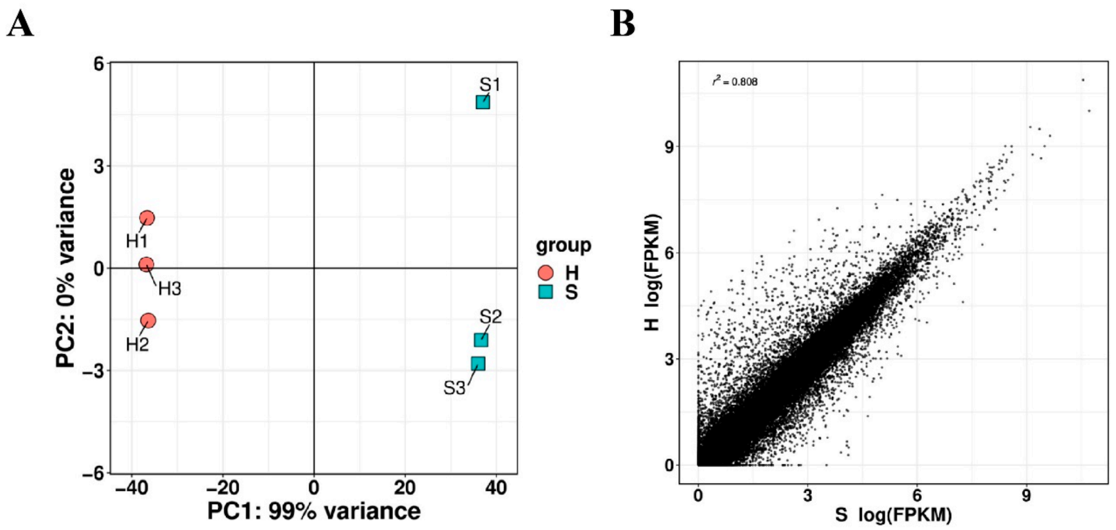

Figure S2 PCA and correlation analysis of transcriptome data. A. PCA analysis of transcriptome data. B. Correlation analysis of transcriptome data.

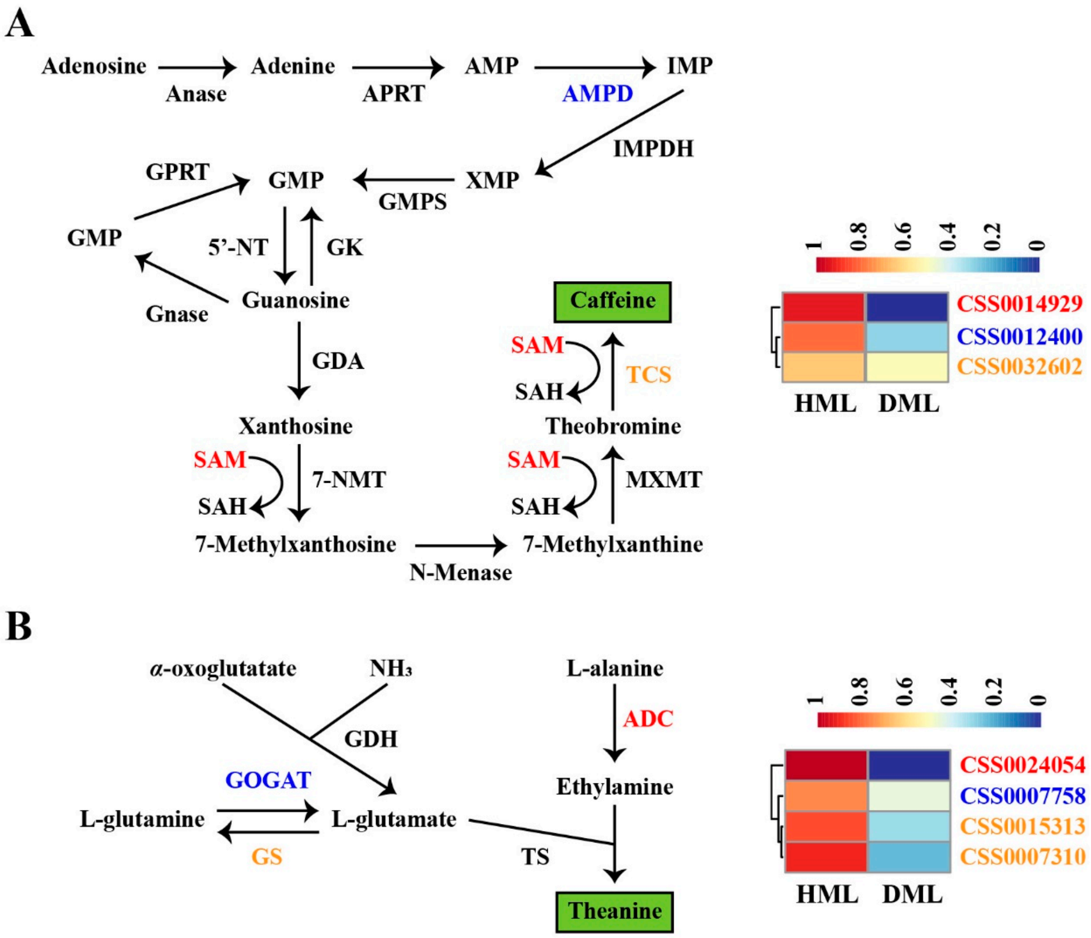

Figure S3 Expression analysis of DEGs involved in caffeine and theanine biosynthesis pathways in the mature leaves of healthy and diseased tea plants. A. Expression analysis of DEGs involved in caffeine biosynthesis pathway in the mature leaves of healthy and diseased tea plants. B. Expression analysis of DEGs involved in theanine biosynthesis pathway in the mature leaves of healthy and diseased tea plants. The compound names were shown below each arrow. The abbreviations beside the arrows indicated the enzymes catalysing the transfer. HML, healthy mature leaves; DML, diseased mature leaves.
