# Supplementary material for: Study on the Effect of Sooty Mould Disease in Tea Plants
Source: Plants (Basel). 2024 Aug 20;13(16):2321. doi: 10.3390/plants13162321 (PMC11359017; doi:10.3390/plants13162321)
Supplement: Supplementary file 1 [file plants-13-02321-s001.zip › Supplementary Table S.pdf]

Table S1 Information of the species gene used for phylogenetic analysis

Species	Isolate/culture collection	GenBank accession number		
		ITS	TEF1	ACT
Cladosporium pini-ponderosae	CBS: 124456	FJ936160.1	FJ936164.1	FJ936167.1
Cladosporium cucumerinum	CBS: 171.52	HM148072.1	HM148316.1	HM148561.1
Cladosporium antarcticum	CBS: 690.92	EF679334.2	EF679405.1	EF679484.1
Cladosporium basi-inflatum	CBS: 822.84	HM148000.1	HM148241.1	HM148487.1
Cladosporium cladosporioides	CBS: 112388	HM148003.1	HM148244.1	HM148490.1
Cladosporium guizhouense	COAD_3377	——	OQ605780.1	OQ605789.1
Cladosporium guizhouense	GUCC: 401.8	ON334728.1	ON383470.1	ON383338.1
Cladosporium pseudocladosporioides	KUC1671	JN033475.1	JN033530.1	JN033502.1
Cladosporium cladosporioides	CPC: 18230	JF499834.1	JF499872.1	JF499878.1
Cladosporium pseudocladosporioides	UTHSC: DI-13-232	LN834382.1	LN834478.1	LN834566.1
Cladosporium pseudocladosporioides	MUT1384	KF415079.1	——	KF415078.1
